# Dystrophin and calcium current are decreased in cardiomyocytes expressing Cre enzyme driven by αMHC but not TNT promoter

**DOI:** 10.1038/s41598-019-55950-w

**Published:** 2019-12-19

**Authors:** Ludovic Gillet, Sabrina Guichard, Maria C. Essers, Jean-Sébastien Rougier, Hugues Abriel

**Affiliations:** 1 Ion Channels and Channelopathies Laboratory, Institute for Biochemistry and Molecular Medicine, University of Bern, Bern, Switzerland; 2 Present Address: Roche Diagnostics International Ltd., Rotkreuz, Switzerland

**Keywords:** Cell biology, Molecular biology

## Abstract

The Cre/lox system is a potent technology to control gene expression in mouse tissues. However, cardiac-specific Cre recombinase expression alone can lead to cardiac alterations when no loxP sites are present, which is not well understood. Many loxP-like sites have been identified in the mouse genome that might be Cre sensitive. One of them is located in the *Dmd* gene encoding dystrophin, a protein important for the function and stabilization of voltage-gated calcium (Ca_v_1.2) and sodium (Na_v_1.5) channels, respectively. Here, we investigate whether Cre affects dystrophin expression and function in hearts without loxP sites in the genome. In mice expressing Cre under the alpha-myosin heavy chain (MHC-Cre) or Troponin T (TNT-Cre) promoter, we investigated dystrophin expression, Na_v_1.5 expression, and Ca_v_1.2 function. Compared to age-matched MHC-Cre^−^ mice, dystrophin protein level was significantly decreased in hearts from MHC-Cre^+^ mice of more than 12-weeks-old. Quantitative RT-PCR revealed decreased mRNA levels of *Dmd* gene. Unexpectedly, calcium current (*I*_CaL_), but not Na_v_1.5 protein expression was altered in those mice. Surprisingly, in hearts from 12-week-old and older TNT-Cre^+^ mice, neither *I*_CaL_ nor dystrophin and Na_v_1.5 protein content were altered compared to TNT-Cre^−^. Cre recombinase unpredictably affects cardiac phenotype, and Cre-expressing mouse models should be carefully investigated before experimental use.

## Introduction

Over the past decades, different genome manipulation technologies have been developed to investigate the function of specific genes *in vivo*. The Cre/loxP system has been of particular interest since it offers the possibility to generate tissue-specific and time-dependent knock-in (KI) or knock-out (KO) mice^1,2^. To generate cardiomyocyte-specific KO mice, Cre recombinase is often placed under the α-myosin-heavy chain (MHC) and the Troponin T (TNT) promoters. To date, many murine MHC- and TNT-Cre strains have been generated on different genetic backgrounds. Constitutive Cre expression under the control of MHC promoter can however decrease the cardiac ejection fraction in an age-dependent manner^3^. One potential mechanism to explain the side effects of Cre expression is the presence of loxP-like sites in the wild-type genome that are sensitive to Cre activity^3,4^. One of these loxP-like site is localized in the *Dmd* gene that encodes the structural protein dystrophin^3^. This 427-kilodalton protein is crucial to maintain the integrity of the lateral membrane of cardiac myocytes^5^. The absence of dystrophin in dystrophinopathies such as Duchene muscular dystrophy alters the current mediated by the voltage-gated calcium channel Ca_v_1.2 (*I*_CaL_) and the whole-cell protein content of voltage-gated sodium channel Na_v_1.5^6–9^. Based on these observations and knowing that Ca_v_1.2 and Na_v_1.5 are pivotal players in cardiac function, we compared dystrophin protein expression, *I*_CaL_, and whole-cell Na_v_1.5 protein expression in hearts between MHC-Cre^−^, MHC-Cre^+^, TNT-Cre^−^, and TNT-Cre^+^ mice.

## Results

### Dystrophin expression is reduced in an age-dependent manner in MHC-Cre^+^ mouse hearts

**Figure 1:**
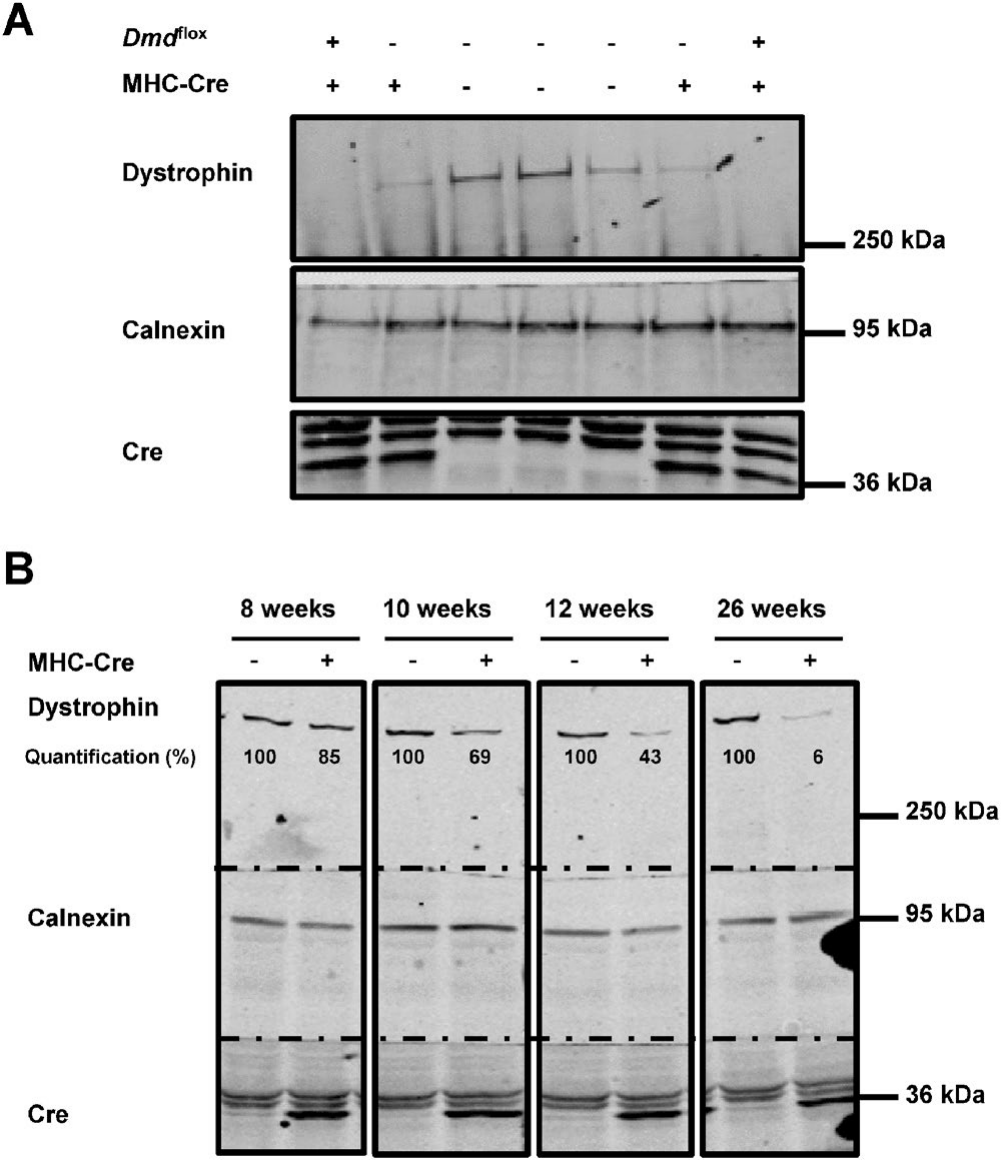
Dystrophin expression is decreased in adult mouse hearts expressing Cre. (**A**) Cropped western blots showing dystrophin expression in the hearts of WT (*Dmd*^flox−^, MHC-Cre^−^), MHC-Cre^+^ (*Dmd*^flox−^, MHC-Cre^+^), and cardiac-specific dystrophin-KO (*Dmd*^flox+^, MHC-Cre^+^) mice. (**B**) Cropped western blots showing dystrophin expression in mouse hearts at different ages with or without Cre recombinase expression. Dystrophin expression is quantified by normalizing band intensity to calnexin band intensity.

**Figure 2:**
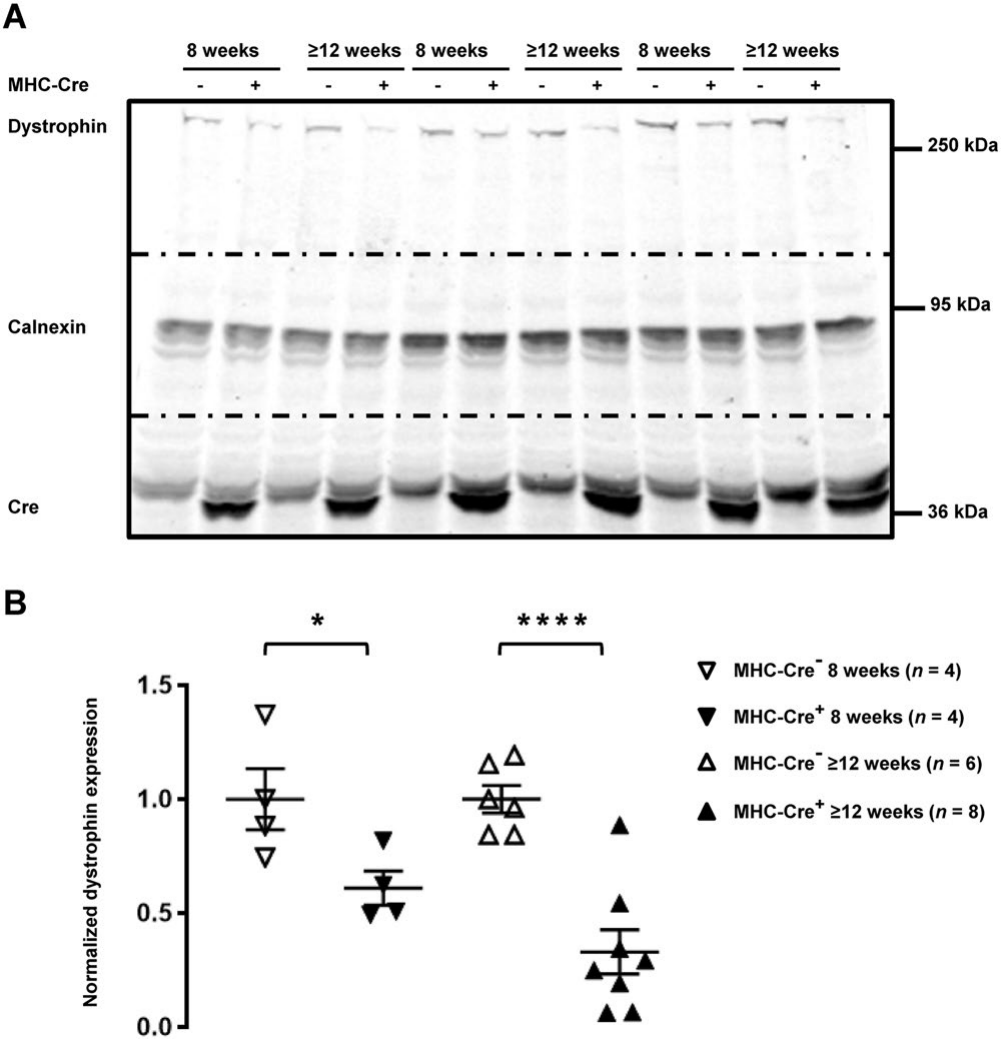
Dystrophin expression is decreased in MHC-Cre^+^ mouse hearts in an age-dependent manner. (**A**) Cropped western blots showing dystrophin expression in MHC-Cre^−^ and MHC-Cre^+^ mouse hearts from 8-week-old and more than 12-week-old (≥12 weeks) mice. (**B**) Quantification of dystrophin expression from 4 to 8 western blots. *p < 0.05 and ****p < 0.0001.

**Figure 3:**
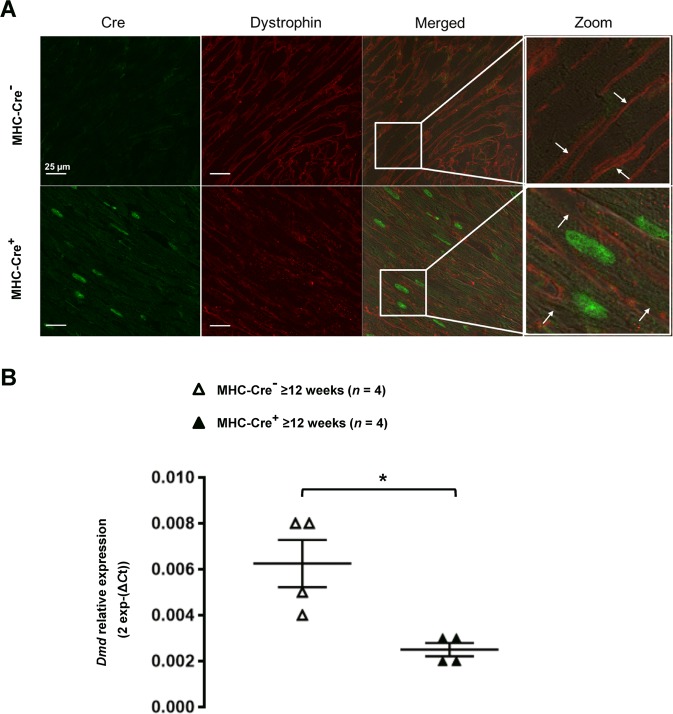
Dystrophin expression is altered in adult MHC-Cre^+^ mouse hearts at protein and mRNA level. (**A**) Representative immunostainings of three independent experiments showing decreased dystrophin signal (red) at the lateral membrane (white arrows), in cardiomyocytes expressing Cre (green). (**B**) Decreased mRNA levels for dystrophin are also observed in adult MHC-Cre^+^ mouse hearts compared with control littermates (MHC-Cre^−^). *p < 0.05.

*Dmd*^flox+^ mice were generated (Suppl. Fig. 1) and used as a control for the Cre recombinase activity under the control of MHC promoter. Western blots were performed using heart lysates from 12-weeks-old wild-type (WT; *Dmd*^flox−^/MHC-Cre^−^), MHC-Cre^+^ (*Dmd*^flox−^/MHC-Cre^+^), and dystrophin KO (*Dmd*^flox+^/MHC-Cre^+^) littermate mice. Dystrophin expression was efficiently suppressed in dystrophin KO compared to WT hearts (Fig. 1A). Interestingly, dystrophin expression was also greatly reduced in the non-floxed MHC-Cre^+^ littermates (Fig. 1A, compare lane 2 and 6 with lane 3–5). In contrast, the presence of the floxed element did not affect the dystrophin expression in heart lysates (Suppl. Fig. 2). To address the time course of dystrophin decrease due to the expression of Cre only, western blots were performed on hearts from 8-, 10-, 12-, and 26-week-old mice. The reduction in dystrophin expression seemed to be more pronounced with age (Fig. 1B). To statistically confirm the age-dependent loss of dystrophin expression, western blots were performed on hearts from 8-, 12-, and ≥12-week-old MHC-Cre^+^ mice and control littermates (MHC-Cre^−^). While dystrophin expression differed only slightly between control and MHC-Cre^+^ hearts at 8 weeks, at ≥12 weeks dystrophin expression was significantly decreased by 70 ± 10% in MHC-Cre^+^ hearts compared to controls (MHC-Cre^−^) (Fig. 2A,B). Immunostainings on cardiac sections from ≥12-week-old MHC-Cre^+^ mouse hearts showed reduced dystrophin expression at the lateral membrane compared to their littermate controls (MHC-Cre^−^) (Fig. 3A).

### *Dmd* mRNA expression is reduced in MHC-Cre^+^ hearts

The reduced expression of dystrophin, leaded us to perform quantitative RT-PCR on mRNAs extracted from ≥12-week-old mouse hearts. The relative decrease of 2^exp−(Δ*CT*)^ measured with MHC-Cre^+^ hearts corresponded to a ~50% reduction in *Dmd* mRNA level in MHC-Cre^+^ compared to the control mice (MHC-Cre^−^) (Fig. 3B). This indicates that the Cre-mediated decrease of dystrophin protein correlates with a decrease of *Dmd* mRNA expression.

### Calcium current *I*_CaL_ but not Na_v_1.5 expression is altered in MHC-Cre^+^ cardiomyocytes

**Figure 4:**
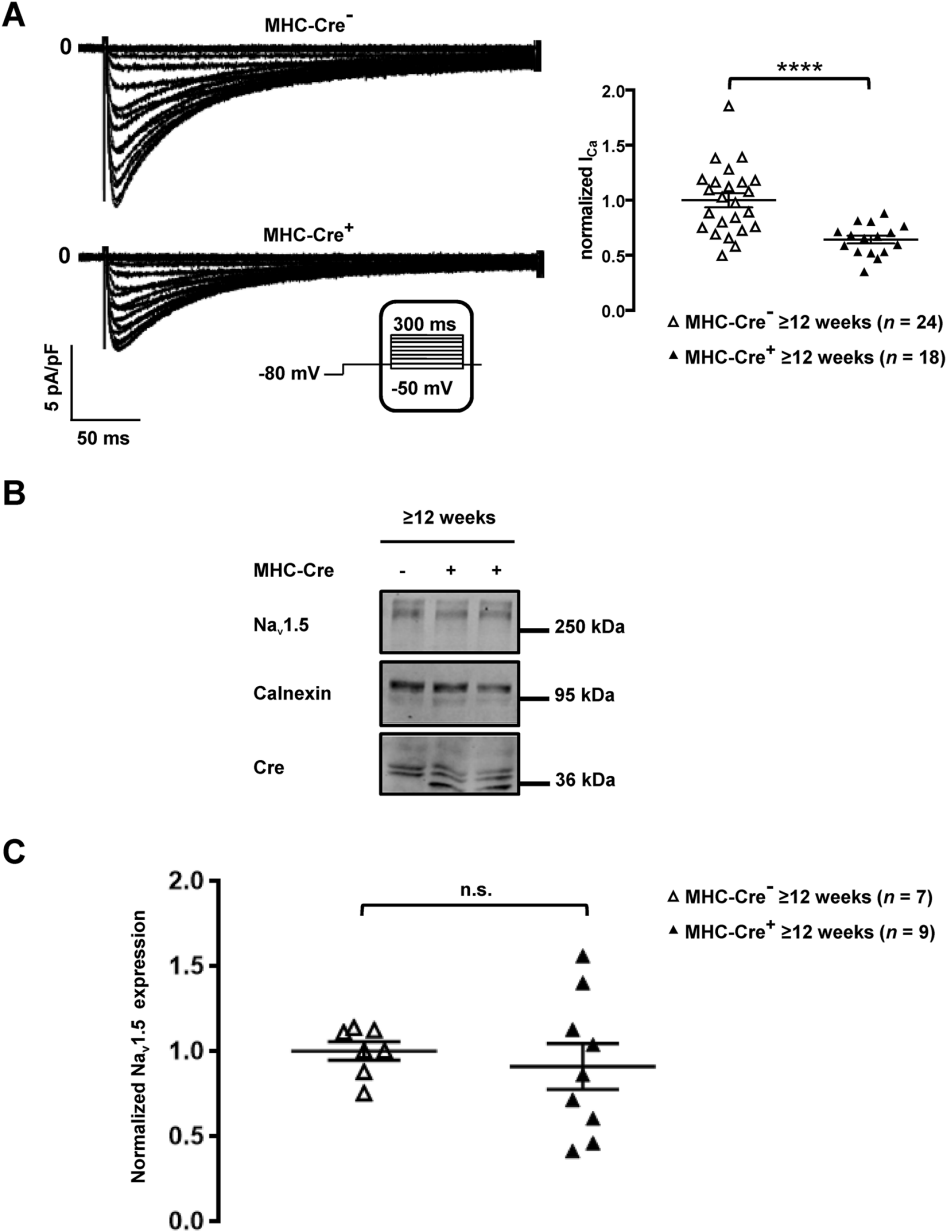
Decreased *I*_CaL_ and unaltered Na_v_1.5 expression in MHC-Cre^+^ mouse cardiomyocytes and mouse hearts respectively. (**A**) Representative traces of *I*_CaL_ recorded in isolated cardiomyocytes (left panel) and quantification of *I*_CaL_ (right panel) shown a significant downregulation of the calcium current in MHC-Cre^+^ cardiomyocytes compared to the control (MHC-Cre^−^) from ≥12-week-old mice. ****p < 0.0001. (**B**) Cropped western blots showing Na_v_1.5 expression in heart lysates from MHC-Cre^+^ and MHC-Cre^−^ ≥12-week^-^old mice. The bottom panel shows the quantification of Na_v_1.5 expression normalized to calnexin expression from 7 to 9 western blots. n.s. indicates a non-statistically significant difference.

Considering the role of dystrophin in the regulation of Ca_v_1.2 currents, we recorded calcium currents in MHC-Cre^+^ isolated cardiomyocytes^9^. Calcium current densities recorded in isolated cardiomyocytes were decreased in cardiomyocytes from ≥12-week-old MHC-Cre^+^ mice compared to controls (MHC-Cre^−^) in contrary to what it has been previously reported^9^ or observed in the dystrophin deficient mice used in this study (Fig. 4A and Suppl. Fig. 3). Since dystrophin regulates sodium channel stability in cardiac myocytes^6,8^, we also studied Na_v_1.5 expression in whole-heart lysates from ≥12-week-old mice. Surprisingly, Na_v_1.5 expression did not differ between MHC-Cre^+^ and MHC-Cre^−^ hearts (Fig. 4B,C).

### No alterations observed in TNT-Cre^+^ mouse line

**Figure 5:**
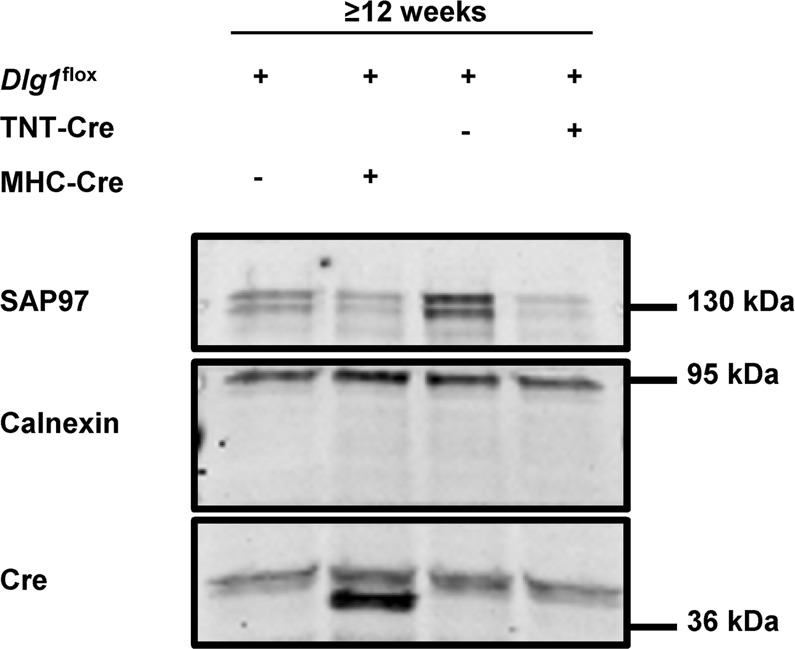
Characterization of adult TNT-Cre^+^ mouse hearts. (**A**) Cropped western blots showing the decrease of expression of SAP97 in transgenic mouse hearts in which SAP97 alleles (*Dlg1*) are floxed (*Dlg1*^flox^) and breed either with TNT-Cre^+^ or MHC-Cre^+^ mice.

**Figure 6:**
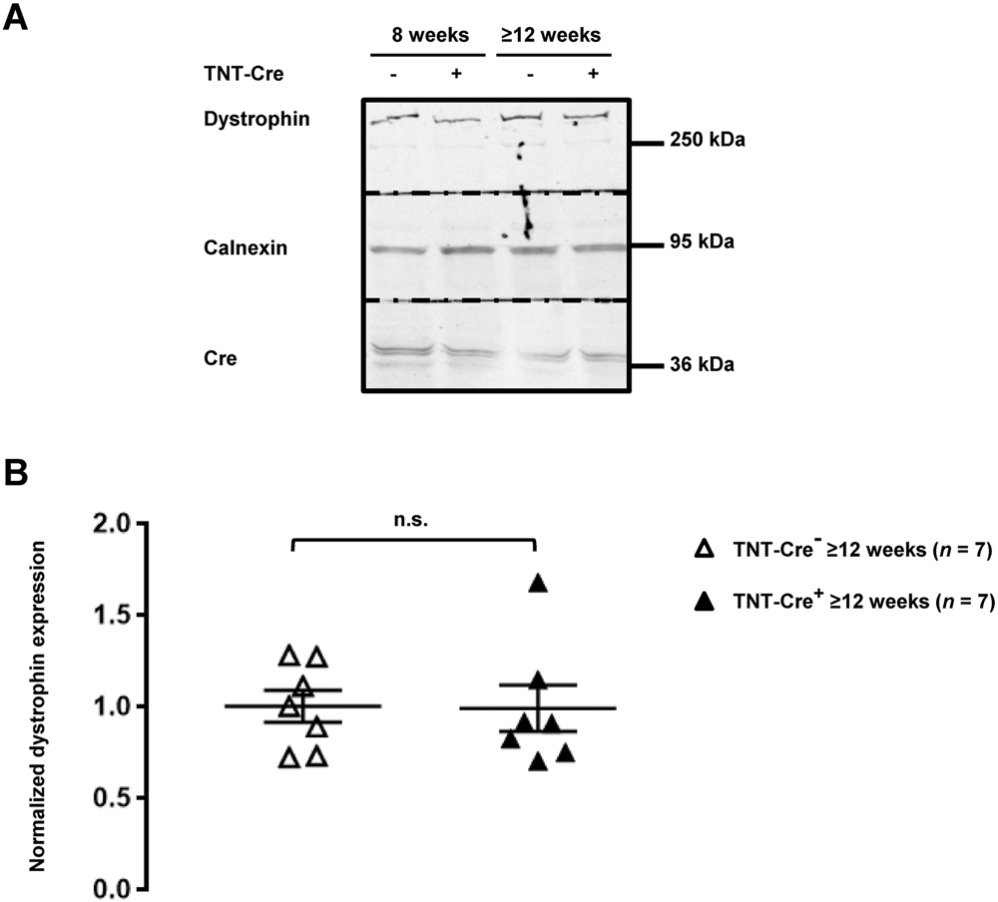
Dystrophin protein expression is not altered in TNT-Cre^+^ mouse hearts. (**A**) Cropped western blots showing dystrophin expression in TNT-Cre^−^ and TNT-Cre^+^ from 8-week-old and 12 or more-week-old (≥12 weeks) mouse heart lysates. (**B**) Quantification of cardiac dystrophin expression from ≥12-week-old mice from 7 western blots. n.s. indicates a non-statistically significant difference.

**Figure 7:**
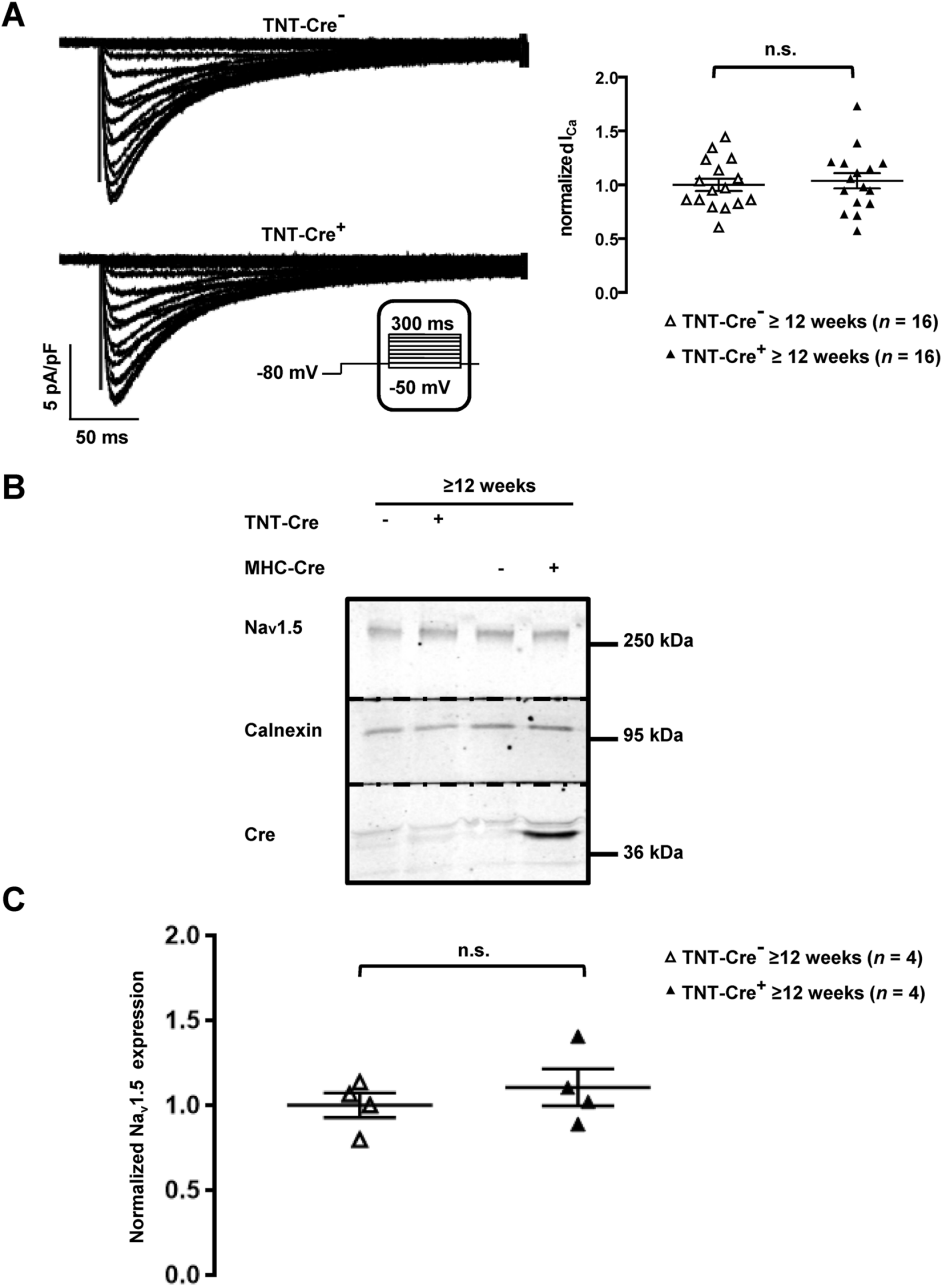
*I*_CaL_ and Na_v_1.5 expression are unaltered in TNT-Cre^+^ mouse cardiomyocytes and mouse hearts. (**A**) Representative traces of *I*_CaL_ recorded in isolated cardiomyocytes (left panel) and quantification of *I*_CaL_ (right panel) show that the calcium current is not changed in TNT-Cre^+^ cardiomyocytes compared to the control (TNT-Cre^−^) from ≥12-week-old mice. n.s. indicates a non-statistically significant difference. (**B**) Cropped western blots showing Na_v_1.5 expression in heart lysates from ≥12-week-old MHC-Cre^+^, MHC-Cre^−^, TNT-Cre^−^, and TNT-Cre^+^ mice. (**C**) Na_v_1.5 expression quantification of 4 western blots normalized to calnexin band intensity. n.s. indicates a non-statistically significant difference.

Based on our observations in MHC-Cre^+^ mouse line, we conducted similar experiments on TNT-Cre^+^ mice, in which Cre recombinase is under the control of the rat troponin T2 cardiac promoter^10^. This strain has been useful for generating early cardiomyocyte-specific mutants since Cre is mainly expressed during embryogenesis^10^. As expected, we observed Cre expression only in new-born TNT-Cre^+^ mouse hearts (Suppl. Fig. 3A,B)^10^. To validate the recombinase activity of the Cre enzyme under the control of the TNT promoter, we crossed TNT-Cre^+^ mice with mice previously characterized *Dlg1*^flox^ mice in which the SAP97-coding gene *Dlg1* was floxed, and compared them to MHC-Cre^+^ *Dlg1*^flox^ mice^11^. SAP97 downregulation was similarly effective in MHC-Cre^+^ and TNT-Cre^+^ hearts from ≥12-week-old mice (Fig. 5). No decrease in dystrophin protein expression was observed in TNT-Cre^+^ hearts from ≥12-week-old mice compared to control littermates (TNT-Cre^−^) (Fig. 6A,B). Moreover, neither calcium currents (Fig. 7A) nor Na_v_1.5 expression (Fig. 7B,C) were altered in cardiomyocytes and heart lysates from ≥12-week-old and more TNT-Cre^+^ mice compared with their respective controls.

## Discussion

MHC-Cre mice have been extensively used to alter the expression of various cardiac genes^12–14^. Our present work however strongly suggests that the MHC-Cre model should be carefully characterized before using it for any experiment, mainly because Cre expression reduces dystrophin protein and mRNA expression. Dystrophin is a crucial protein in many cardiac processes, and dystrophin reduction has been observed in human diseased cardiomyocytes^5^. Secondly, the L-type calcium current density is decreased by about 40% in the MHC-Cre model. Calcium current is pivotal for cardiac excitation-contraction coupling, among other processes, and calcium current dysregulation has been implied in many cardiac arrhythmias^15^.

The observations that protein expression of the sodium channel Na_v_1.5 is not affected in MHC-Cre^+^ mice, although dystrophin-deficient *mdx*^5*cv*^ mice show an overall reduction of Na_v_1.5 expression is unexpected^6^. These discrepancies may be explained by the fact that dystrophin expression is only reduced from 12 weeks of age in MHC-Cre^+^ mice whereas dystrophin is constitutively absent in *mdx*^5*cv*^ mice.

In contrary to the dystrophin-deficient *mdx* mice showing an upregulation of the calcium current^9^, in MHC-Cre^+^ mice, the reduction of dystrophin expression correlates with a decrease of *I*_CaL_. In addition calcium current recordings using the dystrophin deficient mice presented in this study did not reproduced the aforementioned observations (upregulation of the calcium current^9^) (Suppl. Fig. 4). Although, overall all these data are puzzling, the consequences of the absence of dystrophin on the calcium current is still under debate. The gender, the age and probably the genetic background could explain such observations. For example although Rubi and colleagues have shown no alteration of calcium current in male aged dystrophin deficient mice (>25 weeks old)^16^, Li and co-workers reported an increase of this conductance in mixed-sex aged dystrophin deficient mice (>25 weeks old)^17^. In addition, the discrepancy of effects on calcium current in dystrophin deficient mice between our study and the one from Koenig^9^, using young adult mice (<25 weeks old), could be due to the genetic background of the mice used: C57BL/6J and C57BL/10ScSnJ respectively. Overall these data suggest again the importance of proper controls when working with animals.

These results presented in this study suggest also that the expression of other genes is probably altered in the MHC-Cre model leading to complex and unpredictable side effects.

Surprisingly, we observed that Cre expression under the rat troponin T2 cardiac promoter affected neither dystrophin expression nor the calcium current. Those differences of phenotype between MHC-Cre^+^ and TNT-Cre^+^ mice may be explain by two different hypothesis. The first one could be the difference of genetic background. Indeed, mice on mixed backgrounds seem to be more resistant to developing cardiac dysfunction in response to Cre expression than mice on a pure background^18^. The second hypothesis could be the “time window” of Cre activity. Cre recombinase under the TNT promoter is mainly expressed in new-born mice, whereas in MHC-Cre^+^ mice, Cre is constitutively expressed. It is tempting to hypothesize that the off-target effects of Cre mainly occur when Cre recombinase is constitutively expressed after the birth, however further investigations are required to challenge these hypotheses.

Interestingly, a recent study reported the presence of more than 600 loxP-like sites in the genome of C57Bl/6 mice one of which present in the gene coding for the dystrophin^3^. The decrease of mRNA coding for dystrophin observed in MHC-Cre^+^ mice could be due to the Cre recombinase activity on the dystrophin gene containing the loxP-like site. However further experiments should be performed to validate this hypothesis. Based on the amount of loxP-like sites, we may therefore speculate that other genes and proteins are dysregulated in MHC- and TNT-Cre models, which could have various unexpected effects including those observed in this study. Based on these observations, one hypothesis to explain the decrease of calcium current densities in MHC-Cre^+^ ventricular cardiomyocytes compared to the control may be due to an unspecific effect of the cre recombinase either on the gene coding for the voltage-gated calcium channel or on other genes coding for proteins involved in the regulation of the calcium current. A comprehensive proteomic analysis on MHC-Cre^+^ and TNT-Cre^+^ hearts would be required to address the effect of Cre expression on the non-floxed genome.

Cre expression alone likely induces a disease-like phenotype that may confound the effects of any Cre-mediated gene deletion. Indeed, Buerger *et al*. have observed dilated cardiomyopathy in MHC-Cre^+^ mice, further highlighting a potential deleterious effect of the Cre recombinase under the control of the MHC promoter^14^. Pugach *et al*. also reported that prolonged expression of Cre recombinase decreases cardiac function and leads to DNA damage, fibrosis, and cardiac inflammation in MHC-Cre^+^ mice^3^. Although the constitutive expression of Cre in the MHC-Cre model may underlie the aforementioned problems, alternative models all have sizeable shortcomings. Firstly, the MHC-MerCreMer mouse line allows inducible rather than constitutive cardiac-specific expression of Cre driven by the MHC promoter^19^. MerCreMer is a fusion protein containing Cre recombinase with two modified estrogen receptor ligand-binding domains at both ends. Treatment with oestrogen receptor modulators such as tamoxifen induce Cre expression. Stec *et al*. however reported that systolic function is impaired in a MHC-MerCreMer mouse strain^20^. Subsequently, two groups showed that tamoxifen-induced Cre expression is linked to cardiac fibrosis and DNA damage, leading to heart failure and death^21,22^.

Cre recombinase expression can also be regulated by a reverse tetracycline transactivator that is specifically expressed in the heart^23^. Cre is produced once the Cre expression repressor doxycycline is removed from food and water^23^. However, to our knowledge, the effect of this approach on cardiac function has not been investigated yet.

Recently, Werfel and colleagues established a promising approach for cardiac gene inactivation by transfer of the Cre recombinase gene using adeno-associated viral vectors serotype 9 (AAV9)^24^. This technology may induce cardiac-specific inactivation of the floxed gene without adverse side effects on cardiac function^24^. However, more research is required.

In view of these problems, Olson and colleagues have generated a cardiac-specific transgenic mouse using the Clustered Regularly Interspaced Short Palindromic Repeat (CRISPR)-associated (Cas) 9 approach. Cardiac-specific overexpressing of Cas 9 did not have side effects^25^. Then, they delivered a single-guide RNA to the heart using AAV9. This elegant method may be a better alternative to the Cre/lox system^25^.

In conclusion, the findings of the present study serve as a warning to researchers who use engineered mouse lines. Our results strongly suggest that experimenters should carefully chose the proper controls when using Cre-expressing mouse models to avoid any misinterpretation.

## Methods

Parts of these methods have already been published previously^11,26,27^.

### Animals

All experiments involving animals were performed according to the Swiss Federal Animal Protection Law and have been approved by the Cantonal Veterinary Administration, Bern. This investigation conforms to the Guide for the Care and Use of Laboratory Animals, published by the US National Institutes of Health (NIH publication no. 85-23, revised 1996)^11,27^. All the experiments have been approved by the veterinary office of Bern, Switzerland. (TVB number: BE 28/19). Male mice from four different transgenic mouse lines were used in this study. Firstly, the B6.FVB-Tg(Myh6-cre) 2182Mds/J line, also known as MHC-Cre, in which the cardiac-specific α-myosin-heavy chain (Myh6) promoter drives expression of Cre, was purchased from the Jackson Laboratory (stock no. 011038). Secondly, the STOCK Tg (Tnnt2-cre) 5Blh/JiaoJ line, also called TNT-Cre, where Cre recombinase expression is under the control of the rat cardiac troponin T2 promoter, was purchased from the Jackson Laboratory (stock no. 024240). These mice were bred from a mixed background to C57BL/6J for at least one generation upon arrival at the Jackson Laboratory. Thirdly, the *Dmd*^flox^ and *Dlg1*^flox^ mouse strains have been generated by by PolyGene AG (Rümlang, Switzerland) (Supplementary Fig. 1)^11^.

For all experiments, animals were anesthesised by intraperitoneal injection of ketamine/xylazine solution (200 mg/kg ketamine and 20 mg/kg xylazine) and killed by cervical dislocation.

### Protein extraction and western blot

Whole hearts were extracted from mice, the atria were removed, and ventricles were homogenized in 1 mL lysis buffer (50 mM HEPES, 150 mM NaCl, 1 mM EGTA, 10% glycerol, 1.5 mM MgCl_2_, and Complete^®^ protease inhibitor cocktail (Roche, Basel, Switzerland)) using a Polytron. Triton X-100 in lysis buffer was then added to make a final concentration of 1% (final lysate volume 2 mL). Samples were lysed on a rotating wheel for 1 hour at 4 °C. Soluble fractions of the mouse heart lysate were obtained by subsequent centrifugation at 13,000 rpm for 15 minutes at 4 °C. To be able to load similar protein amounts from each sample on a gel, protein concentrations were measured in triplicate by Bradford assay and extrapolated on a bovine serum albumin (BSA) standard curve. Samples were denatured at 95 °C for 5 minutes prior to gel loading. To analyse and compare protein expression, 80 μg of each ventricular mouse heart lysate sample was loaded on a homemade 1.5 mm-thick 8–15% SDS-PAGE gel for high molecular weight proteins. Gels were run at 150 V for 1 hour and proteins were subsequently transferred to nitrocellulose membranes using the Biorad Turbo Transfer System (Biorad, Hercules, CA, USA). All membranes were stained with Ponceau as a qualitative check for equivalent total protein loading and transfer. Membranes were then rinsed twice with PBS before western blotting using the SNAP i.d. system (Millipore, Zug, Switzerland). Membranes were blocked with 1% BSA (Sigma) for 10 minutes, followed by incubation with primary antibodies for 10 minutes. Membranes were subsequently washed four times in PBS + 0.1% Tween before incubating with secondary fluorescence antibodies for 10 minutes. After four more washes with PBS + 0.1% Tween and three washes in PBS, fluorescently labelled proteins were detected on a Odyssey® Infrared Imaging System (LI-COR Biosciences, Bad Homberg, Germany). The protein content was subsequently quantified by measuring and comparing band densities (equivalent to fluorescence intensities of the bands) using the Image Studio software version 5.2.5^11,26,27^.

### Immunohistochemistry of mouse ventricular section

Following removal of atria, hearts were cut transversally, embedded in Tissue-Tek® O.C.T™ Compound (Sakura Finetek, Zoeterwoude, the Netherlands), and cryo-preserved. Frozen 10 μm-thick sections were stored at −80 °C until later use. Sections were then thawed to room temperature for 30 minutes and subsequently fixed in ice-cold acetone for 10 minutes. Samples were dried at room temperature for 10 minutes. After rinsing in PBS, sections were blocked in PBS with 0.5% Triton X-100, 1% BSA, and 10% normal goat serum for 30 minutes. Sections were rinsed again in PBS before addition of primary antibodies (in PBS with 0.5% Triton X-100, 1% BSA, and 3% normal goat serum). After 2 hours of incubation, sections were rinsed again in PBS and then incubated for 1 hour with secondary antibodies. After a final wash with PBS, FluorSave™ Reagent (Calbiochem, La Jolla, CA, USA) was applied to the sections, which were then stored at 4 °C and analysed on a confocal microscope (Zeiss Z-710)^26^.

### Antibodies

For western blots, a mouse monoclonal anti-dystrophin antibody (NCL-DYS2, Novocastra, Muttenz, Switzerland) was used at a dilution of 1/250. A mouse monoclonal antibody against SAP97 (VAM-PS005F, Enzo life science, Lausen, Switzerland) was used at a dilution of 1/500. Against Na_v_1.5, a custom-made rabbit polyclonal antibody (Pineda Antibody Service, Berlin) was used at a dilution of 1/200. A rabbit polyclonal antibody against Cre (69050; Novagen, EMD Millipore-MERK, Schaffhausen, Switzerland) was used at a dilution of 1/500 and a rabbit polyclonal antibody against calnexin (C4731; Sigma-Aldrich, Saint-Louis Missouri, USA) was used at 1/1000^11,26,27^.

For immunostainings, rabbit polyclonal antibody against Cre (69050; Novagen, EMD Millipore-MERK, Schaffhausen, Switzerland) was used at a dilution of 1/500 and mouse monoclonal anti-dystrophin (Dys NCL-DYS2, Novocastra, Muttenz, Switzerland) was used at a dilution of 1/250^11,26,27^.

### RNA extraction and quantitative RT-PCR

Total RNA was isolated from mouse heart pieces with the ExpressArt RNAready kit (Amp Tec GmbH, Germany, provided by the lab of professor Jaggi, Bern, Switzerland). The High Capacity cDNA Reverse Transcription Kit (Applied Biosystems, Life Technologies, Switzerland) was used to synthesize cDNA. Subsequently, 25 ng of cDNA was mixed with TaqMan Universal Master Mix (Invitrogen) and 1 µL of either dystrophin (*Dmd*) or glyceraldehyde-3-phosphate dehydrogenase (GAPDH) probe/primer mix (Applied Biosystems; Mm00464475_m1 and Mm99999915_g1, respectively). GAPDH was used as the reference gene to which experimental data was normalized. All samples were loaded in quadruplicates. On an ABI 7500 RT-PCR machine, samples were run with the following thermal cycling conditions: holding stage at 95 °C for 20 seconds, then 40 cycles of 95 °C for 3 seconds and 60 °C for 30 seconds. Relative *Dmd* expression (ΔCT) was calculated by subtracting the signal threshold cycle (CT) of the control (GAPDH) from the CT value of *Dmd*. Results were then linearized by calculating 2^exp−(*ΔCT*)^^11^.

### Isolation of mouse ventricular myocytes

Single cardiomyocytes were isolated according to a modified procedure of established enzymatic methods. Briefly, mice were euthanized by cervical dislocation. Hearts were rapidly excised, cannulated and mounted on a Langendorff column for retrograde perfusion at 37 °C. Hearts were rinsed free of blood with a nominally Ca^2+^-free solution containing (in mM): 135 NaCl, 4 KCl, 1.2 MgCl_2_, 1.2 NaH_2_PO_4_, 10 HEPES, 11 glucose, pH 7.4 (NaOH adjusted), and subsequently digested by a solution supplemented with 50 µM Ca^2+^ and collagenase type II (1 mg/mL, 300 U/mg, Worthington, Allschwil, Switzerland) for 15 minutes. Following digestion, the atria were removed and the ventricles transferred to nominally Ca^2+^-free solution, where they were minced into small pieces. Single cardiac myocytes were liberated by gentle trituration of the digested ventricular tissue and filtered through a 100 µm nylon mesh. Ventricular mouse cardiomyocytes were used after an extracellular calcium increase procedure to avoid calcium overload when applying extracellular solutions in electrophysiology assays^11,26,27^.

### Electrophysiology

Whole-cell currents were measured at room temperature (22–23 °C) using a VE-2 amplifier (Alembic Instrument, USA). The internal pipette solution was composed of (in mM) 60 CsCl, 70 Cs-aspartate, 1 MgCl_2_, 10 HEPES, 11 EGTA and 5 Mg-ATP, pH 7.2, with CsOH. The external solution contained (in mM) 130 NMDG-Cl, 5 CsCl, 2 CaCl_2_, 1.2 MgCl_2_, 10 HEPES and 5 D-glucose, pH 7.4, with CsOH. Data were analysed using pClamp software, version 10.2 (Axon Instruments, Union City, California, USA). Calcium current densities (pA/pF) were calculated dividing the peak current by the cell capacitance^11,26,27^.

### Statistical analyses

Data are represented as means ± S.E.M. Statistical analyses were performed using Prism7 GraphPad^™^ software. Mann-Whitney two-tailed U test was used to compare two groups. One-way ANOVA followed by Sidak’s multiple comparisons test was used to compare more than two groups. *p* < 0.05 was considered significant.

## Supplementary information

Supplementary information

## Data Availability

All data underlying the results are available as part of the article and no additional source data are required.
