# Supplementary material for: Dystrophin and calcium current are decreased in cardiomyocytes expressing Cre enzyme driven by αMHC but not TNT promoter
Source: Sci Rep. 2019 Dec 19;9:19422. doi: 10.1038/s41598-019-55950-w (PMC6923407; doi:10.1038/s41598-019-55950-w)
Supplement: Supplementary file 1 — Supplementary information [file 41598_2019_55950_MOESM1_ESM.pdf]

SUPPLEMENTARY INFORMATION

Dystrophin and calcium current are decreased in cardiomyocytes expressing Cre enzyme driven by αMHC but not TNT promoter

Short title: αMCH-Cre reduces dystrophin expression and calcium current

Ludovic Gillet[1,2], Sabrina Guichard[1], Maria C. Essers[1], Jean-Sébastien Rougier[1], and Hugues Abriel[1]

[1], Ion Channels and Channelopathies Laboratory, Institute for Biochemistry and Molecular Medicine, University of Bern, Switzerland.
[2], Current address: Roche Diagnostics International Ltd., Rotkreuz, Switzerland.

Correspondence should be addressed to:
Prof. Dr. Hugues Abriel, University of Bern, Institute of Biochemistry and Molecular Medicine
Bühlstrasse 28, 3012, Bern, Switzerland
hugues.abriel@ibmm.unibe.ch
Phone number: +41 31 631 31 86

Supplemental Figure 1. Generation of the $\mathit{Dmd}^{\mathrm{flox}}$ mouse model. A) The homology regions in the wild type (WT), the targeted allele (TG) and the targeted allele after deletion of neomycin cassette (TGdelneo) are indicated as grey boxes. The elongated homology (SA elongated 2) is depicted with a white box. Exons are depicted with black arrows. After homologous recombination, two loxP sites (grey arrows) are inserted into the genome flanking exon 22 of *Dmd*. Additionally, an FRT flanked neomycin cassette (dashed white arrow) is inserted downstream of exon 22. This cassette is deleted upon Flp-mediated recombination, leaving only the proximal loxP and a single FRT site (dashed white arrow) downstream of exon 22.

Supplemental Figure 2. The presence of flox elements in $\mathit{Dmd}^{\mathrm{flox}}$ mouse hearts does not alter the dystrophin expression. A) Cropped western blots showing the dystrophin expression in ≥12-week-old mouse heart lysates of $\mathit{Dmd}^{\mathrm{flox}}$ mice with Cre recombinase (MHC-Cre^{+}) or without (MHC-Cre^{-}). N.B: The “cre blot” shown is similar that the one presented in figure 4B because the samples loaded are identical and only one blot against Cre protein has been performed to confirm the presence or not of the Cre recombinase.

Supplemental Figure 3. I_{CaL} are unaltered in dystrophin deficient mouse cardiomyocytes. A) Quantification of I_{CaL} show that the calcium current is not changed in dystrophin deficient cardiomyocytes compared to the wild-type from ≥12-week-old mice. n.s. indicates a non-statistically significant difference.

Supplemental Figure 4. In mouse hearts, Cre recombinase under the control of TNT promoter is expressed mainly in new-born mice. A) Cropped western blots showing the Cre expression in new-born TNT-Cre mouse heart lysates. B) Cropped western blots showing

the absence of Cre recombinase expression in TNT-Cre^+ heart lysates of ≥12-week-old mice compared to MHC-Cre^+ hearts.

Data Availability

“All data underlying the results are available as part of the article and no additional source data are required.”

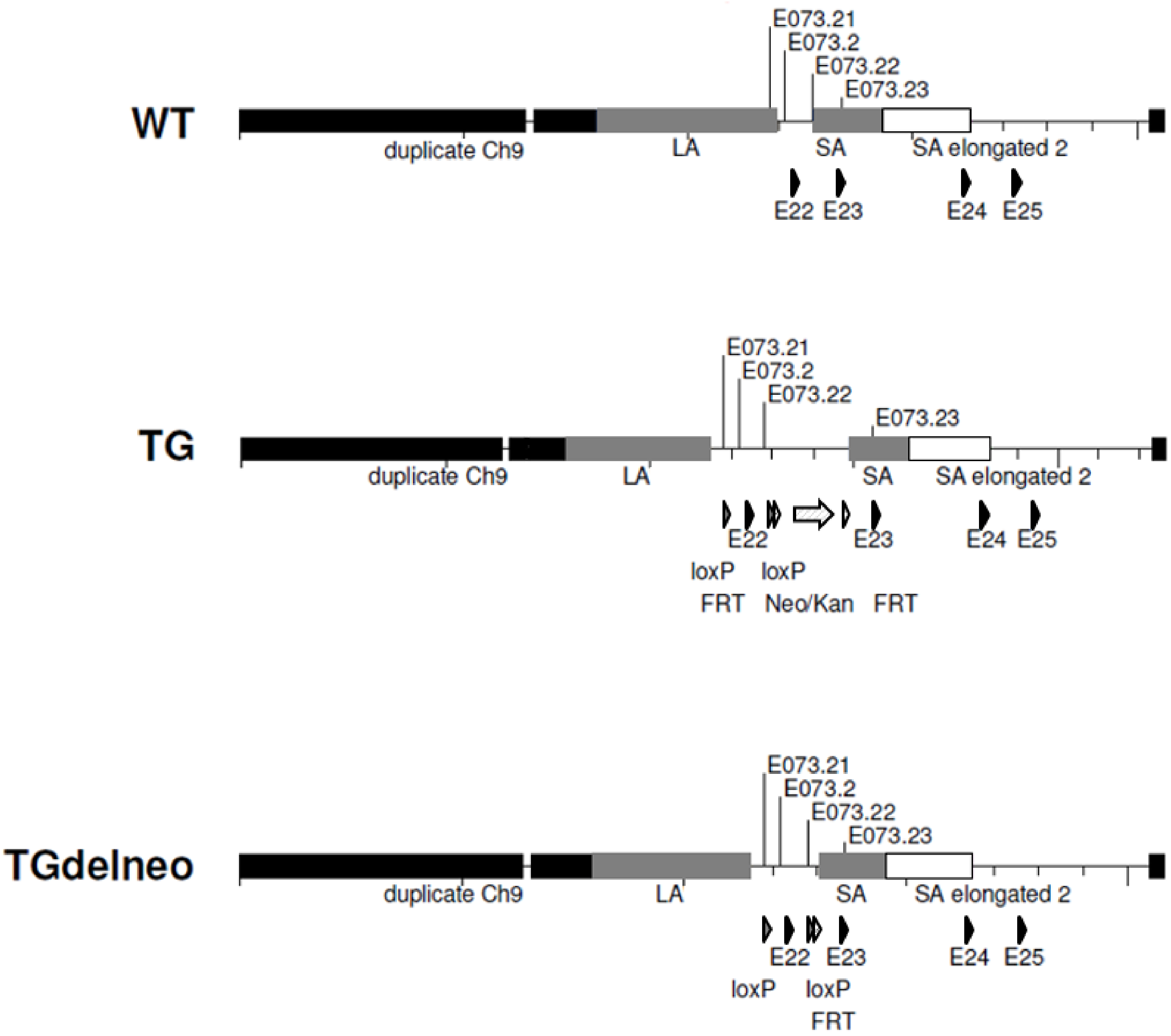

Supplemental Figure 1

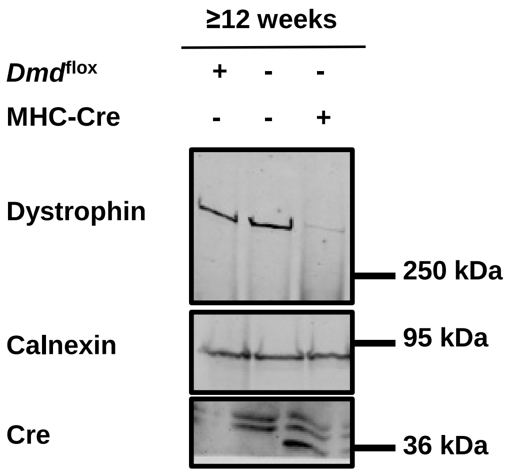

Supplemental Figure 2

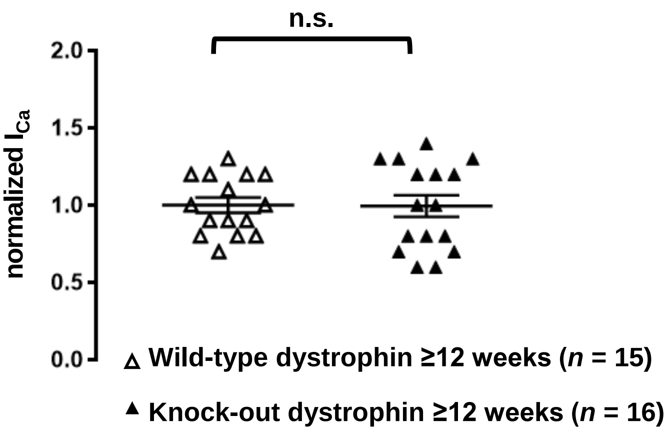

Supplemental Figure 3

A

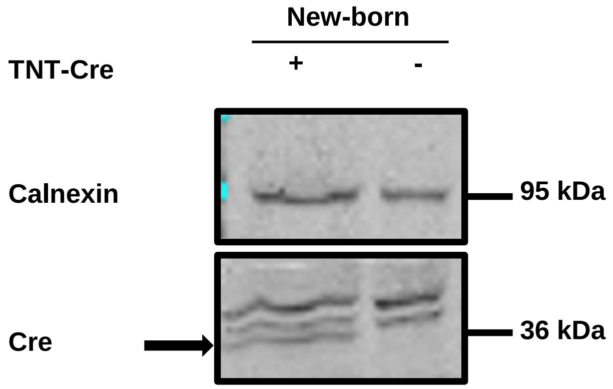

B

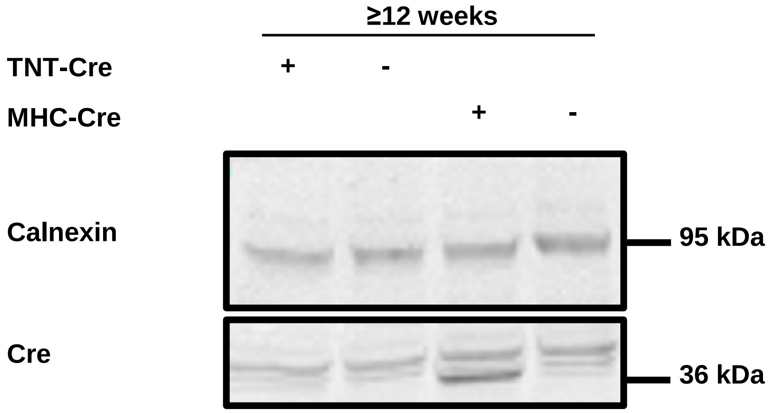

Supplemental Figure 4

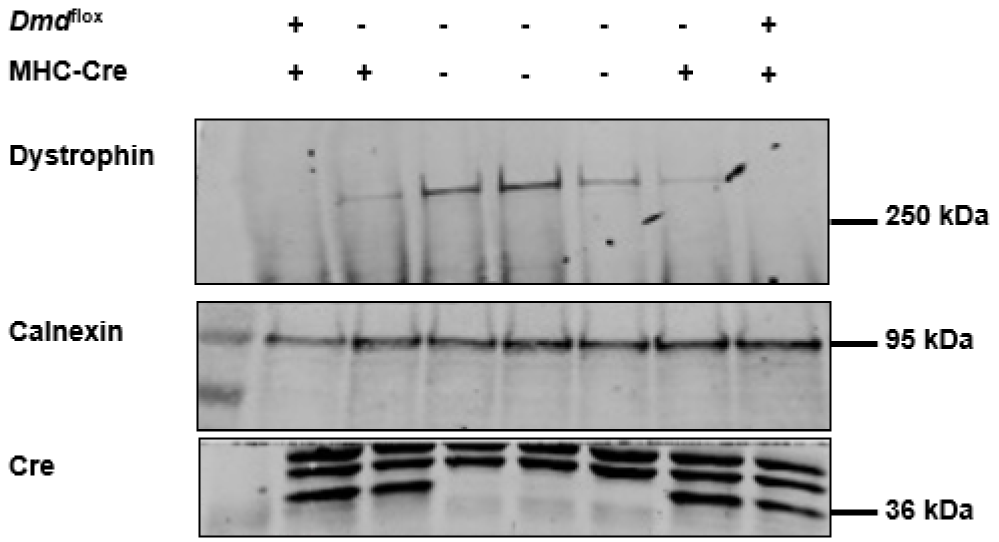

Raw western blot from Figure 1A

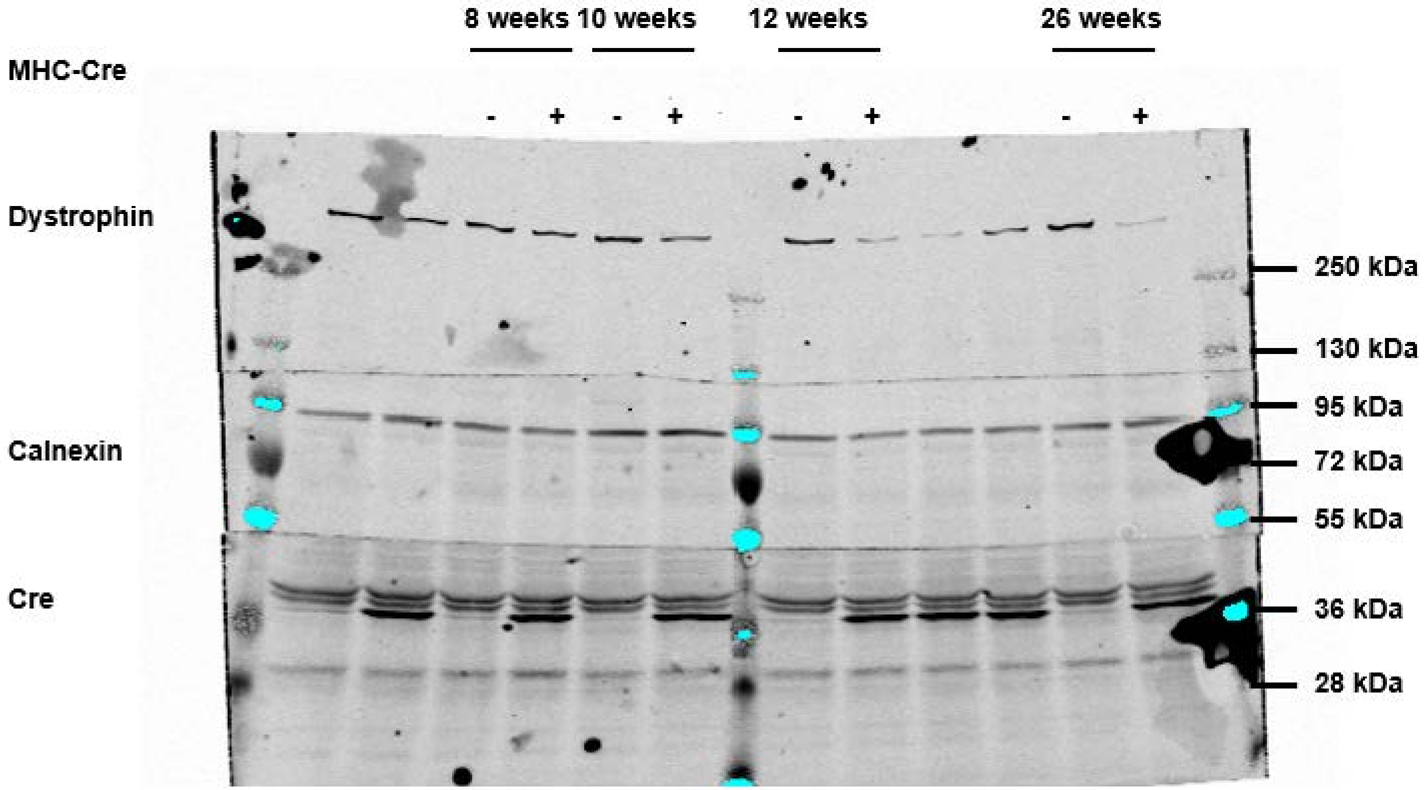

Raw western blot from Figure 1B

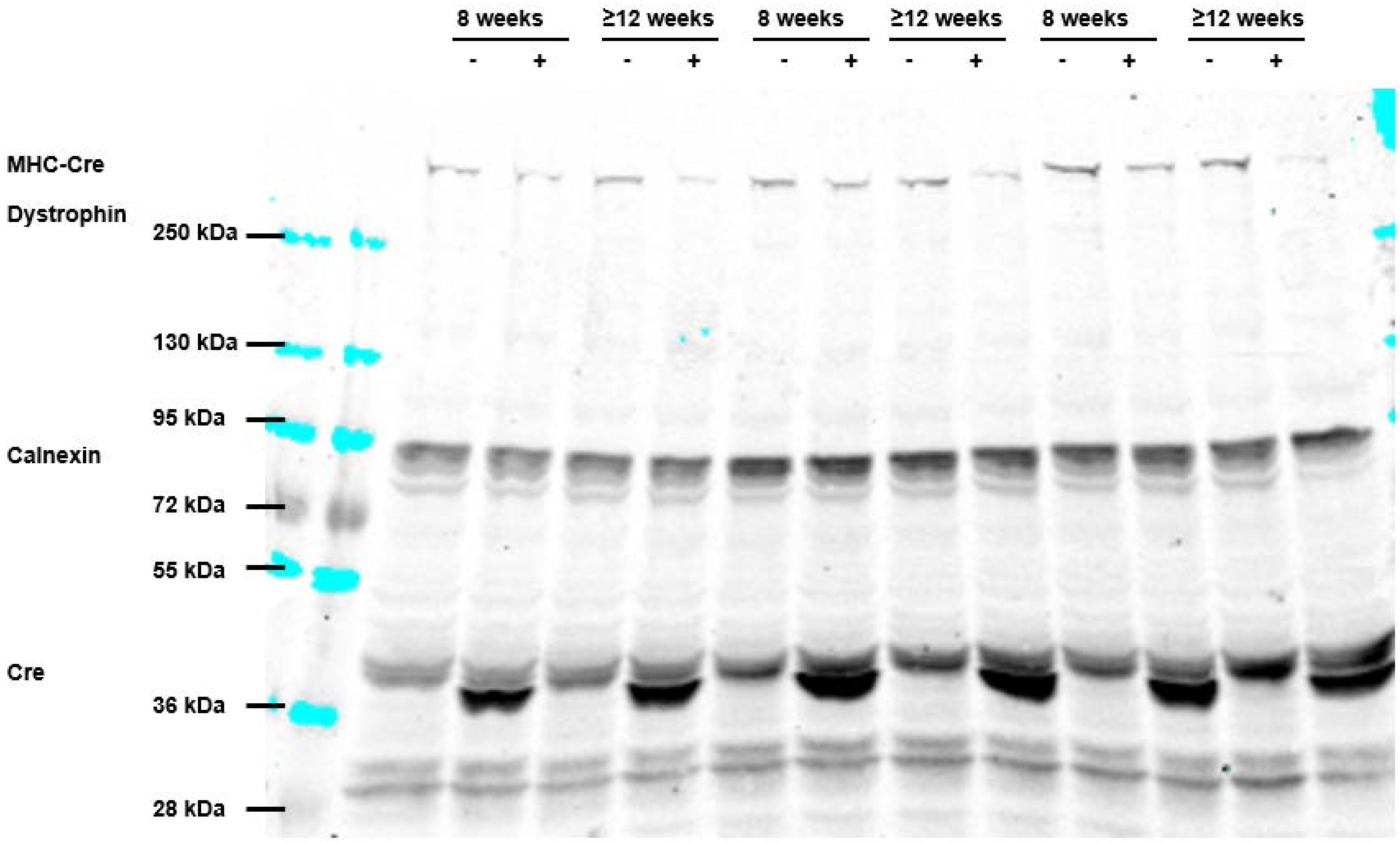

Raw western blot from Figure 2A

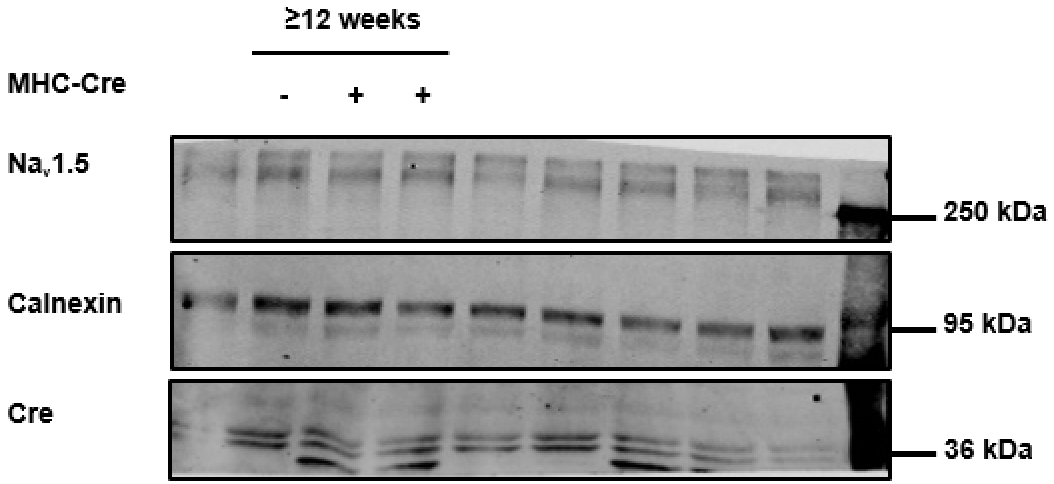

Raw western blot from Figure 4B

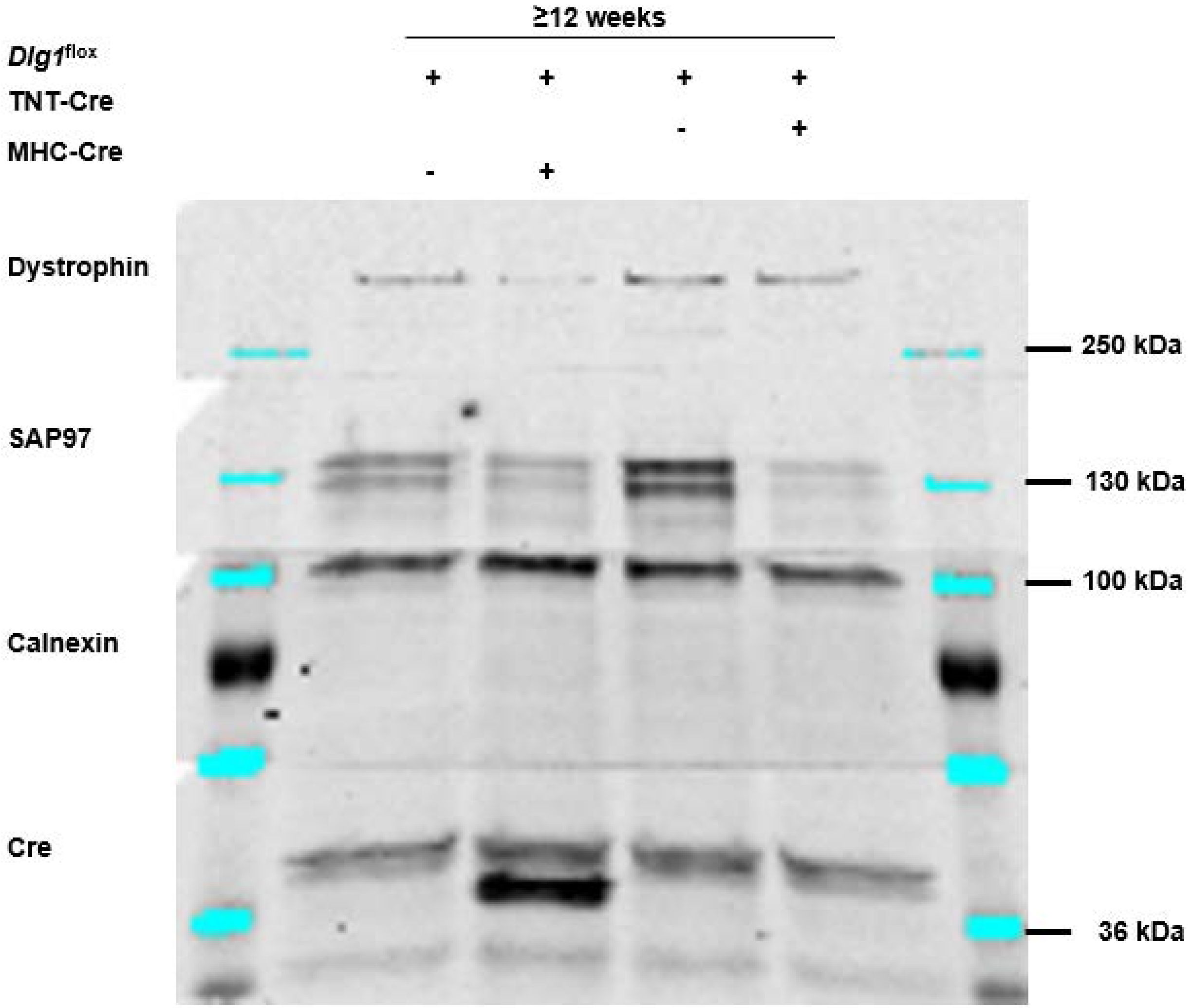

Raw western blot from Figure 5

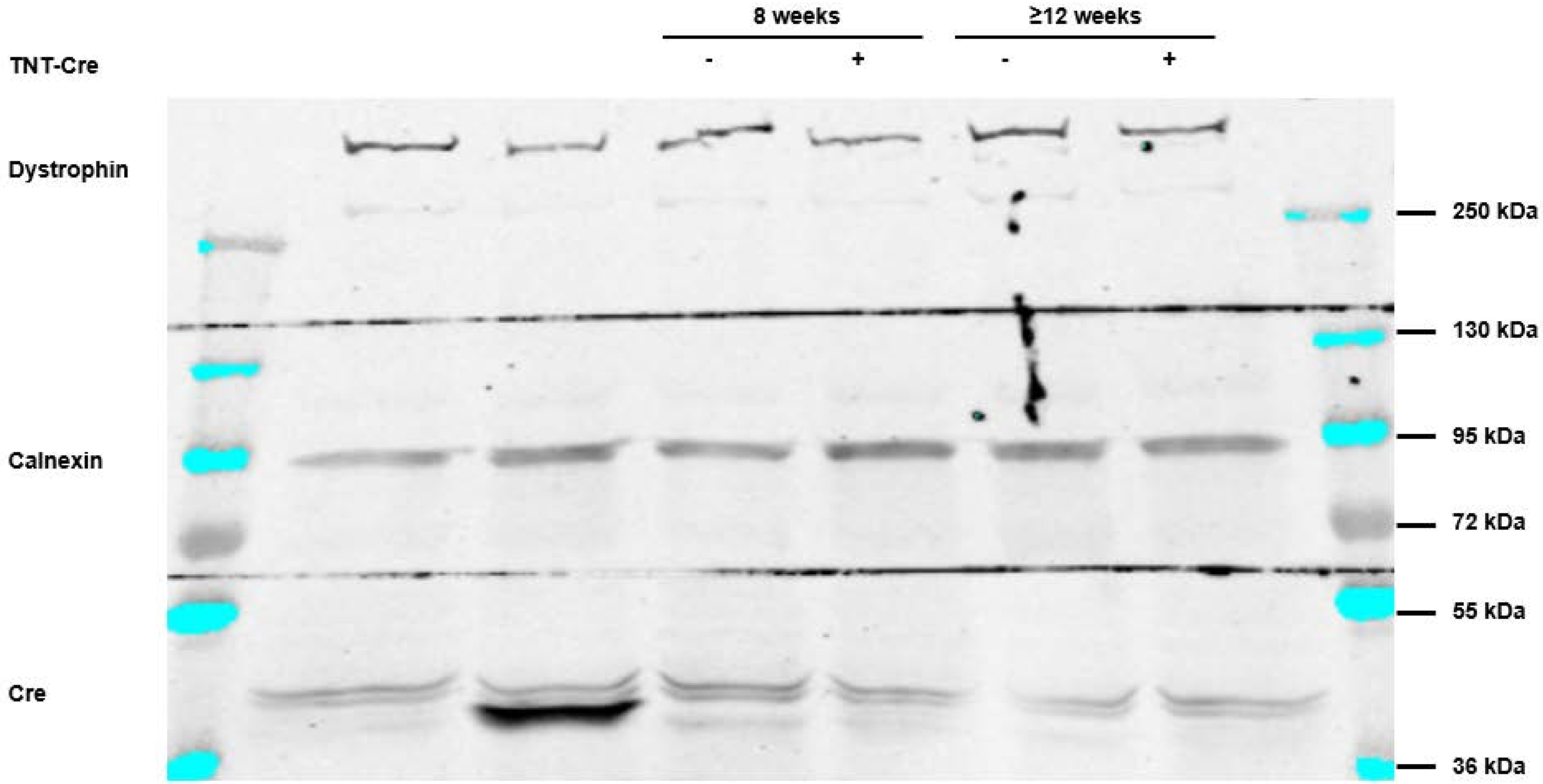

Raw western blot from Figure 6A

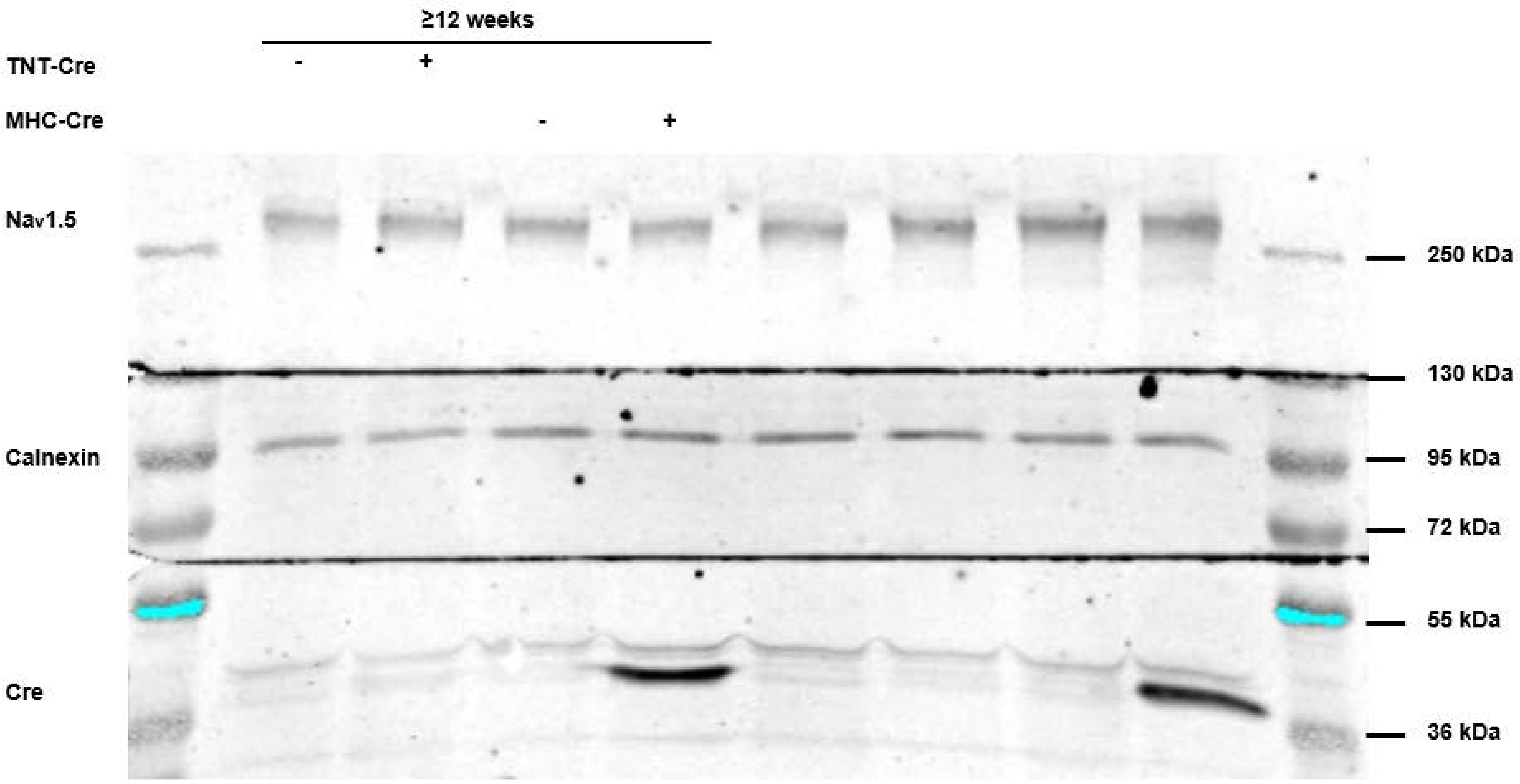

Raw western blot from Figure 7B

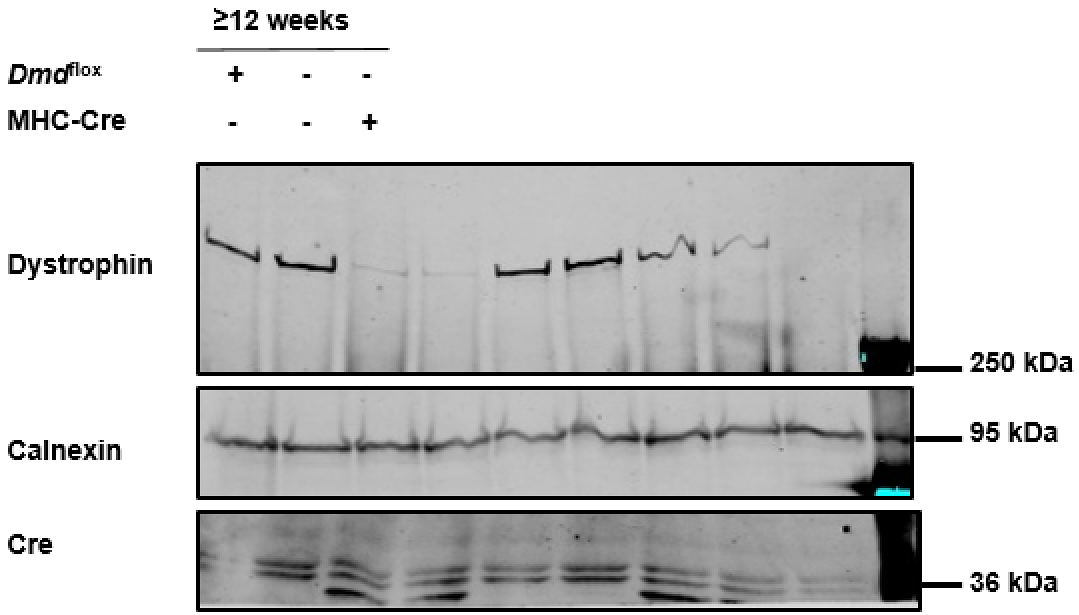

Raw western blot from Supplementary Figure 2

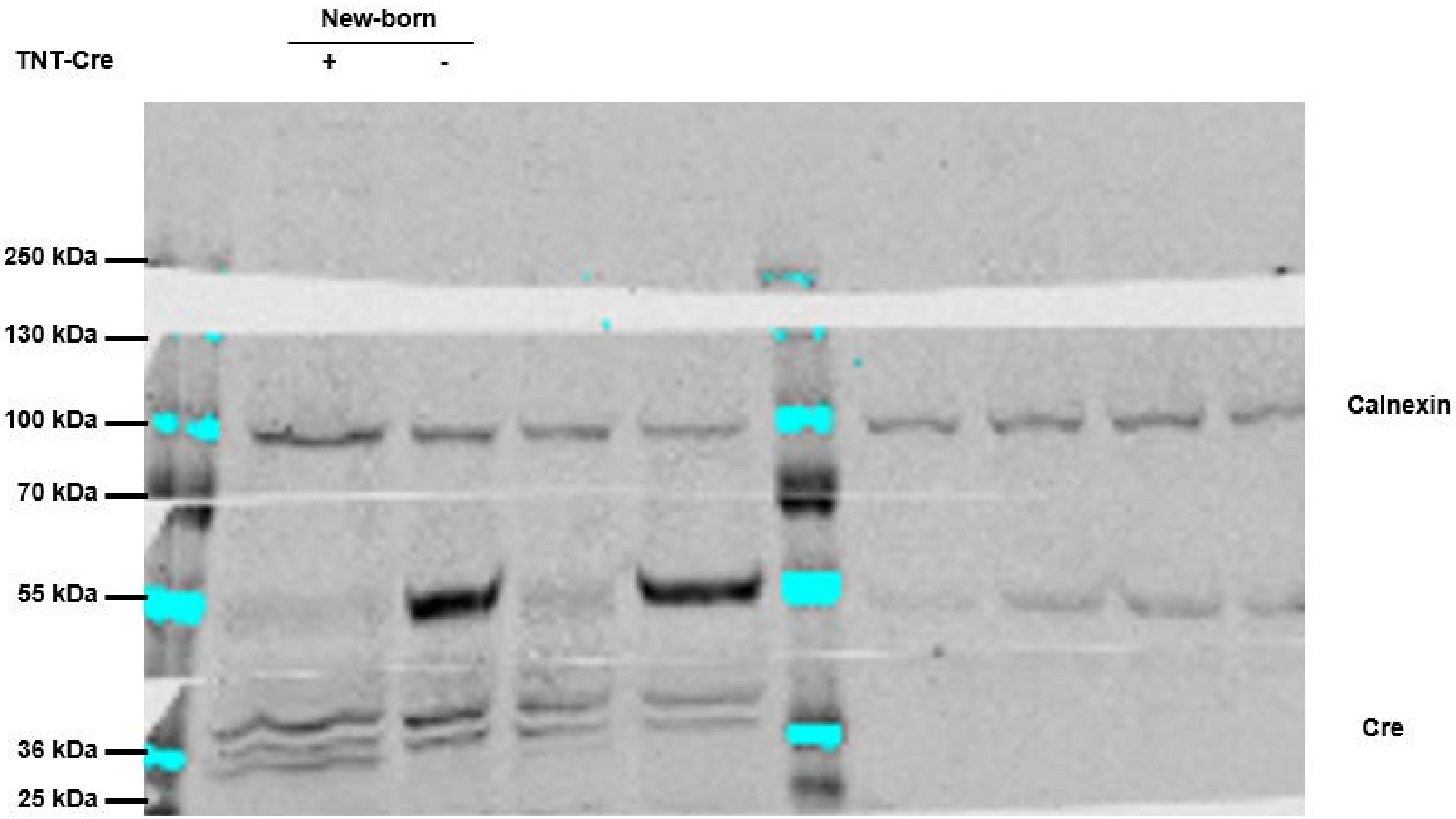

Raw western blot from Supplementary Figure 4A

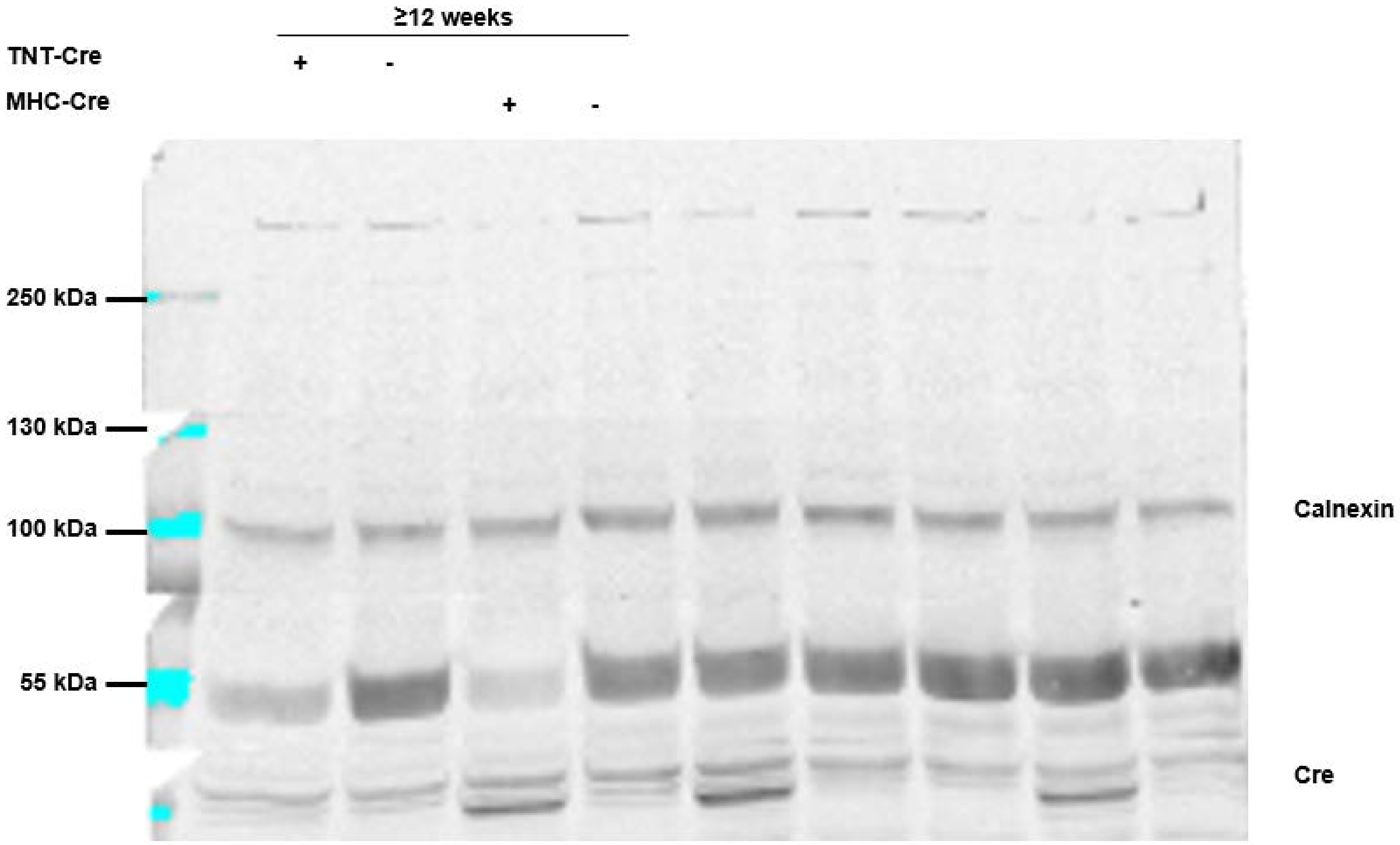

Raw western blot from Supplementary Figure 4B

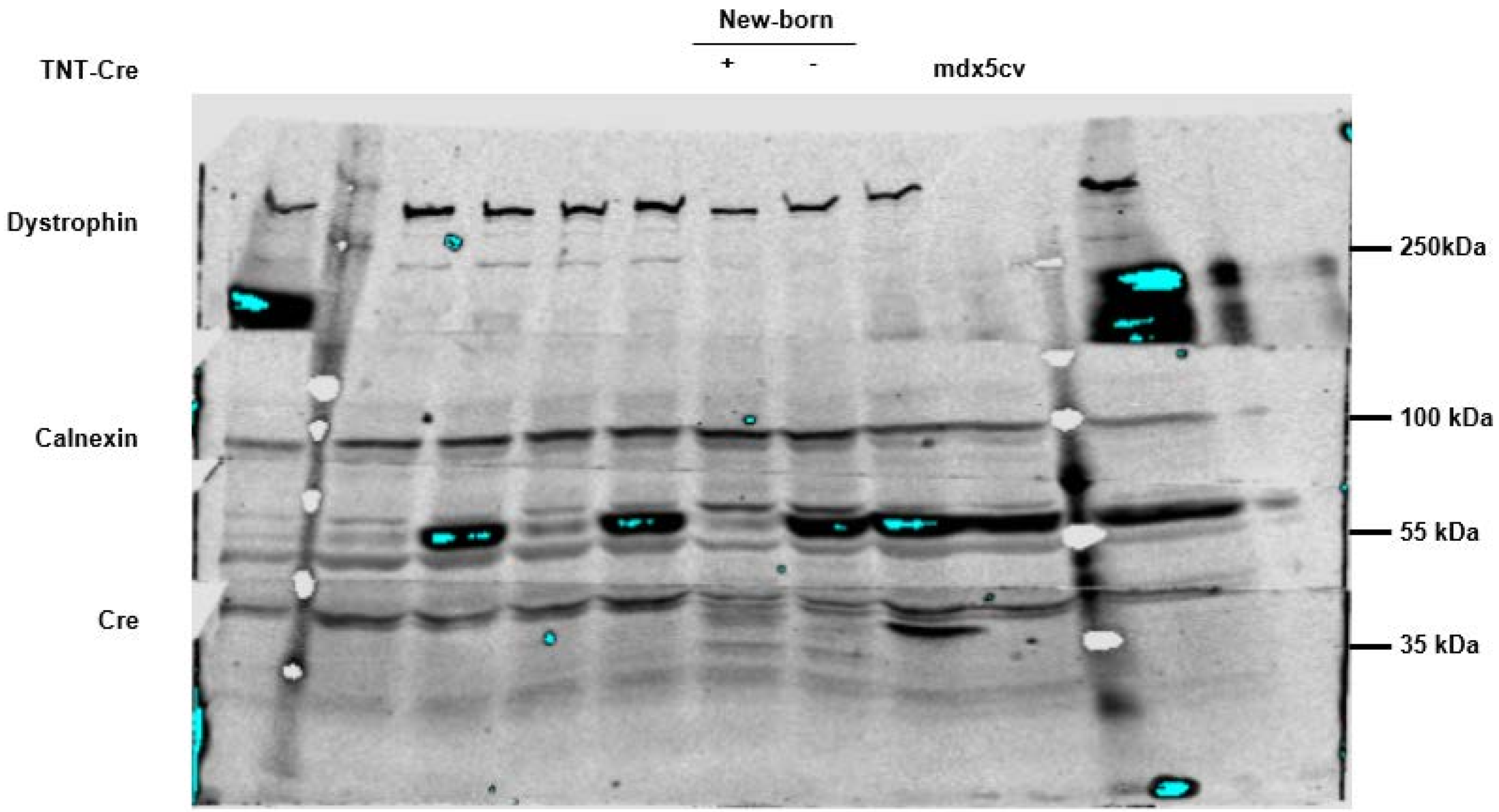

Raw western blot for the reviewer

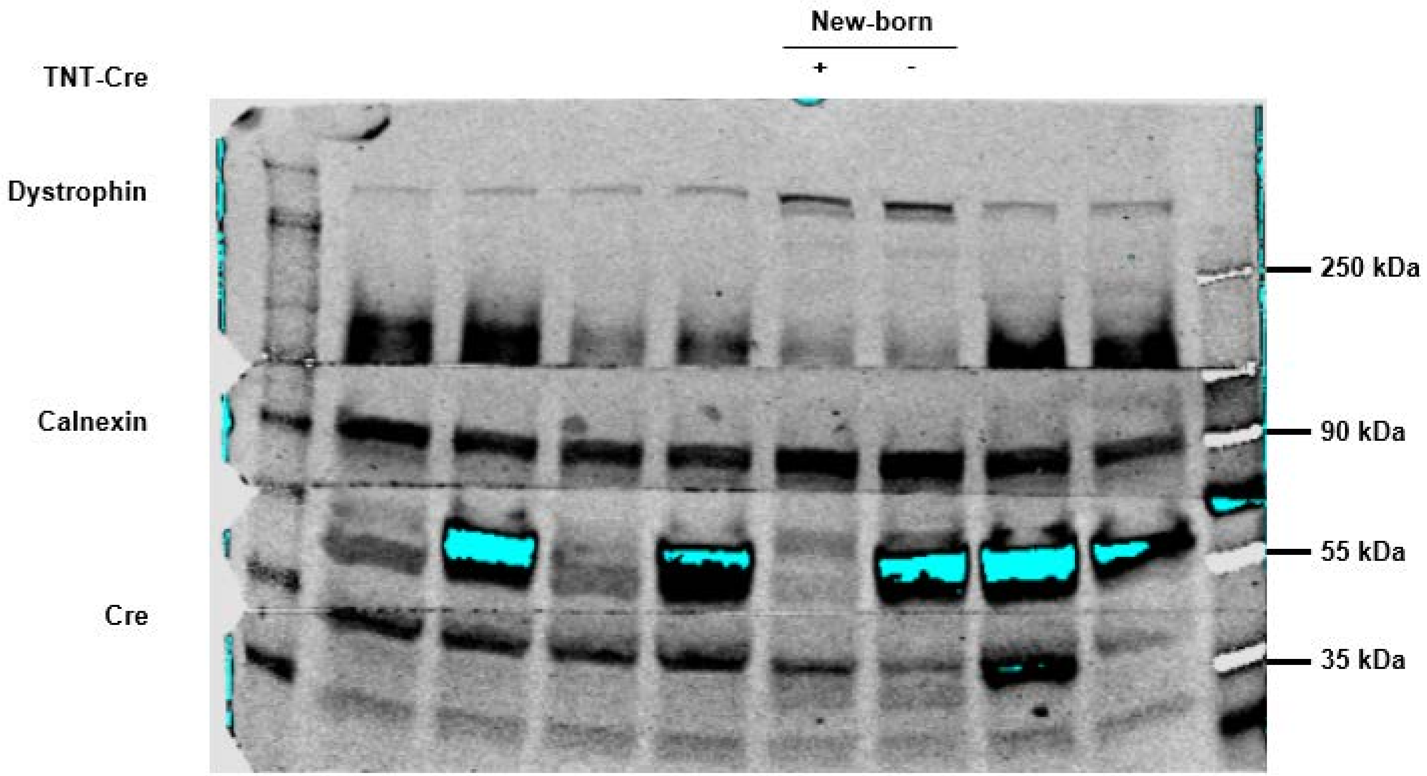

Raw western blot for the reviewer
